# Current Management of Paediatric Supracondylar Fractures of the Humerus

**DOI:** 10.7759/cureus.8137

**Published:** 2020-05-15

**Authors:** Pritom M Shenoy, Amirul Islam, Rahul Puri

**Affiliations:** 1 Trauma and Orthopaedics, Wrexham Maelor Hospital, Wrexham, GBR; 2 Trauma and Orthopaedics, Wythenshawe Hospital, Manchester, GBR; 3 Trauma and Orthopaedics, Apollo Hospitals, Bangalore, IND

**Keywords:** supracondylar humeral fracture, elbow injury, humeral fracture, fracture in a child, cubitus varus, elbow trauma

## Abstract

Supracondylar fractures of the humerus in children are common and can be distressing injuries to the child, the parents and to the surgical team. Type 1 fractures are managed non-operatively, however displaced fractures (Types 2, 3 and 4) are usually managed surgically. Accurate and repetitive neuromuscular assessment is critical not just for medicolegal reasons but also to expedite management with different specialists if needed. The Rock, paper, scissor, OK technique is simple which is easily understood by most children. We discuss the current evidence with regards to pin diameter, number, pin configuration along with a simple algorithm on how to manage a child with a displaced supracondylar fracture with no pulse focussing mainly on the extension-type fracture.

## Introduction and background

Supracondylar fractures are common elbow injuries affecting children. Its incidence in literature is reported to be between 3.3% and 16.6% [1,2]. It causes a lot of anxiety to all people involved - the patient, the parents and the surgical team as well. These injuries can cause significant complications and morbidity if not treated properly. Controversy exists with regards to size and number of pins and pin configuration. BOAST guidelines (British Orthopaedic Association Standards of Trauma) attempt to standardize assessment and treatment of these injuries [3].

We aim to provide an overview of supracondylar fractures in children and their management as per the current evidence.

## Review

Incidence

They commonly occur between two and ten years of age with equal predilection for boys and girls [4]. They are mostly closed injuries and have associated neuropraxia in 11.3% of fractures [5]. In extension type fractures, which accounts for nearly 98% of all supracondylar fractures, the anterior interosseous nerve is most commonly injured. This is proposed to be firstly due to direct contusion of the dorsal part of the median nerve by the proximal fracture fragment where the anterior interosseus nerve (AIN) fascicles lie. Secondly, the AIN has less ability to stretch since it is fixed when it lies on the interosseous membrane in the forearm by multiple fibrous bands [6]. The median, radial and ulnar nerves are also injured in decreasing frequency.

In flexion type fractures, the ulnar nerve is most commonly injured [5]. Most neurologic deficits identified at the time of injury are temporary and spontaneously recover within six months [7]. However, new neurologic deficit after surgery may need consideration for exploration to ensure the nerve is not trapped within the fracture site. One must also be careful to protect the ulnar nerve when using medial K-wires.

Clinical assessment

This can sometimes be difficult to perform when children are in pain and quite anxious. Analgesia should be provided and adopting a playful and friendly approach helps during neurovascular assessment. This assessment should be repeated at regular intervals and is especially important before and after application of a splint.

Every hospital should have a standardized way of assessment which helps not only improve documentation and practice but also minimizes medicolegal issues should any arise later on. The sensory examination should be done first to gain the confidence of the child since this is unlikely to provoke pain. Autonomous zones of the nerves should be checked to avoid confusion. The pulp of the index finger should be checked for the median nerve. The pulp of the little finger should be checked for the ulnar nerve and the dorsum of the first web space checked for the radial nerve.

**Figure 1:**
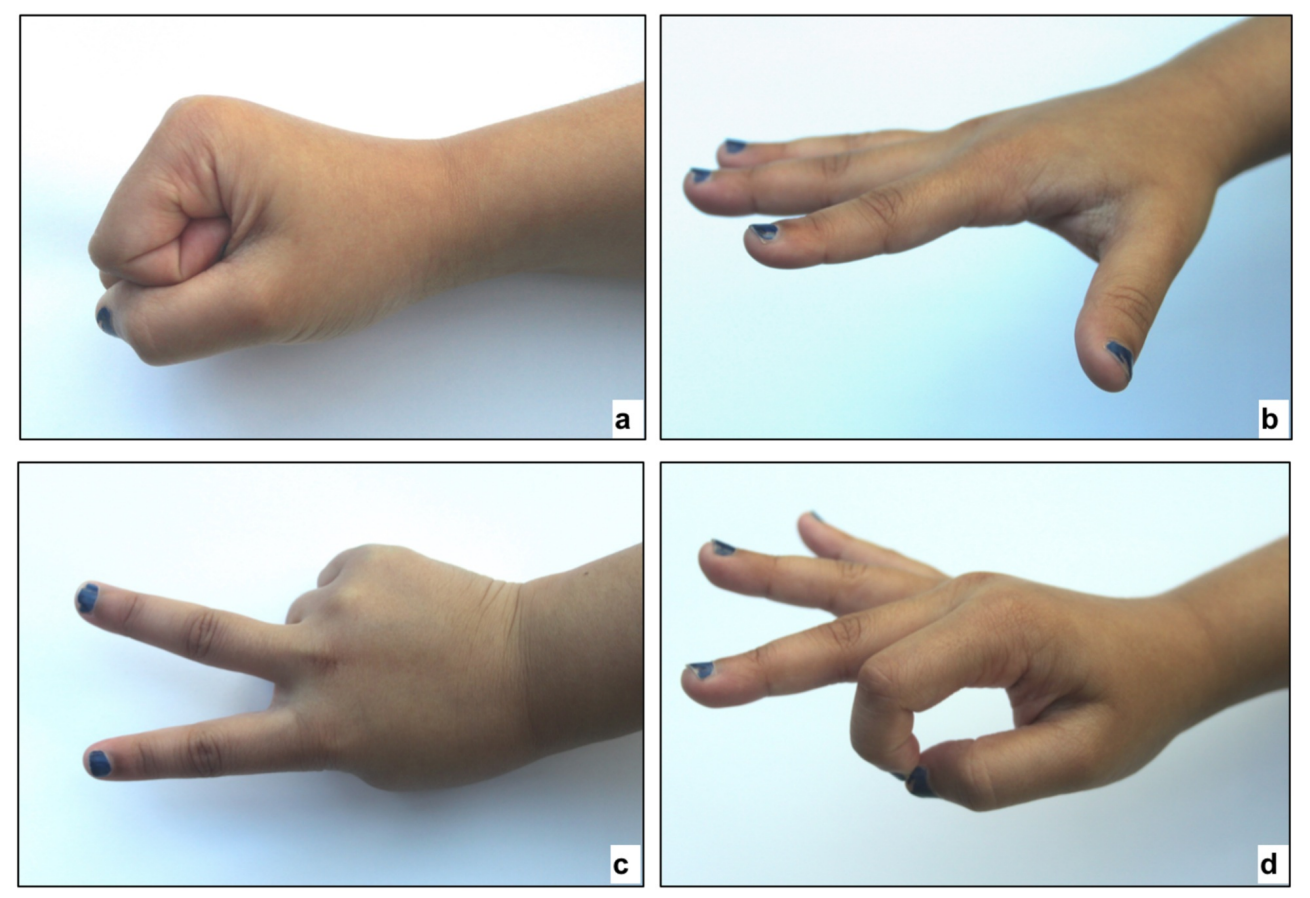
Motor testing using 'Rock, paper, scissors, OK' (a) Rock tests the median nerve. (b) Paper tests the radial nerve. (c) Scissors tests the ulnar nerve. (d) OK tests the anterior interosseous nerve. (Photo courtesy P. M. Shenoy)

Assessing motor function using the “Rock, paper, scissor, OK” approach is simple [8]. As shown in Figure 1, ‘rock’ tests the median nerve, ‘paper’ tests the radial nerve, ‘scissors’ tests the ulnar nerve and ‘OK’ tests the anterior interosseus nerve.

Accurate vascular assessment of the limb is extremely critical. Radial pulse must be assessed by palpation or in some instances by Doppler ultrasonography. An absent radial pulse may be due to vasospasm of the brachial artery or injury or kinking over the fracture spike. Limb perfusion must be assessed which may be present even in the absence of a radial pulse due to abundant collateral circulation. Limb perfusion is determined by assessing skin colour, temperature and digital capillary refill time. In a limb where the perfusion is compromised, ischaemic symptoms such as increased pain, paraesthesia, reduced temperature, delayed or absent capillary refill time or loss of motor function may be seen and must be dealt with urgently as a surgical emergency. Presence of median or anterior interosseous nerve palsy along with an absent pulse could indicate injury to the brachial artery due to close proximity of these structures [9].

Hence the limb without a radial pulse is either a pink and pulseless hand or a white and pulseless hand. This differentiation is crucial since immediate management is dependent on it.

In certain cases, puckering of the anterior skin is seen. This is also called the brachialis sign and indicates that the distal end of the proximal fragment has button-holed through the brachialis and now lies subcutaneously. There is a high risk of neurovascular injury in these cases and closed reduction is generally difficult. These are cases which need careful assessment and urgent management.

Classification

**Figure 2:**
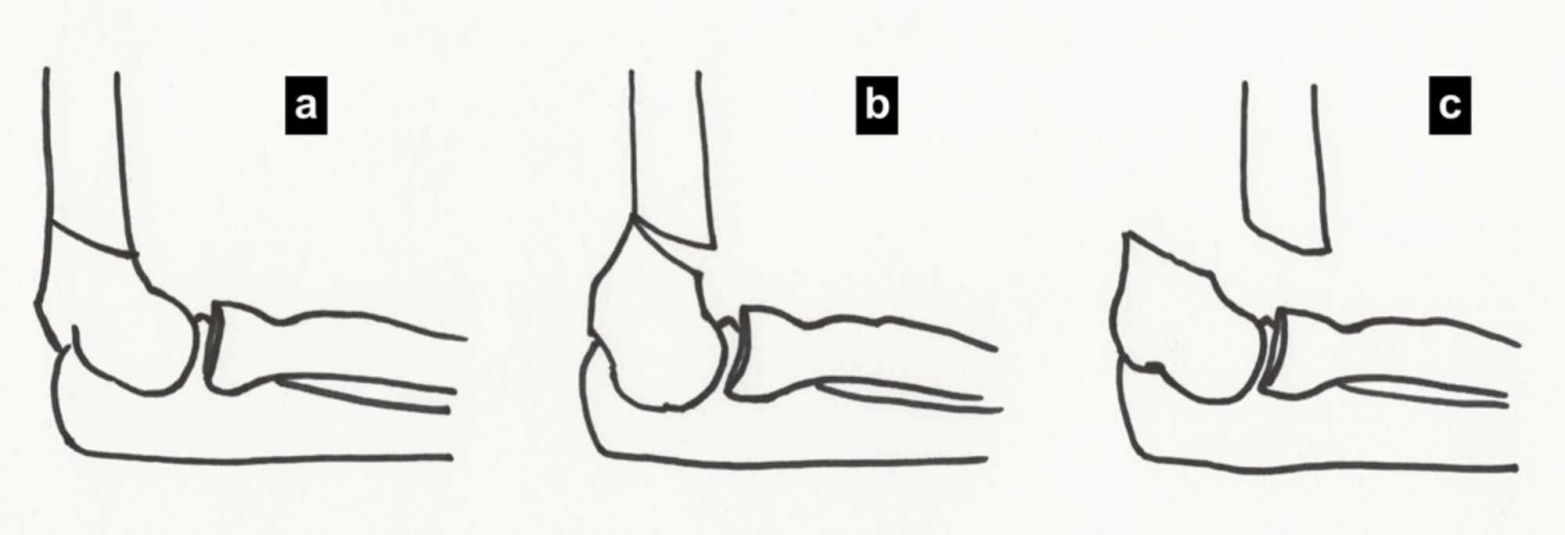
Gartland classification of supracondylar fractures in children (a) Type 1, (b) Type 2, and (c) Type 3. (Illustration courtesy P. M. Shenoy)

Supracondylar fractures are broadly classified into flexion and extension type injuries based on the direction of displacement of the distal fragment. The Gartland classification is commonly used to classify extension type supracondylar fractures (Table 1, Figure 2) [10].

**Table 1:** Gartland classification of supracondylar elbow fractures

Gartland Classification of Supracondylar Elbow Fractures	
		Type 1	Undisplaced fractures
Type 2	Displaced fractures with an intact posterior hinge		
		Type 3	Completely displaced fractures

Wilkins modification of the Gartland classification is mentioned in Table 2 [11].

**Table 2:** Wilkins modification of Gartland's classification

Wilkins Modification of Gartland's Classification	
		Type 1	Undisplaced fractures
Type 2a	Intact posterior cortex with angulation only		
		Type 2b	Intact posterior cortex with angulation and rotation
Type 3a	Displaced in posteromedial direction		
		Type 3b	Displaced in posterolateral direction
Type 4	Displaced and unstable in both flexion and extension		

Radiographic findings

With the advent of digital radiographs, certain features become clearer. The standard X-rays needed are an anteroposterior view with the elbow kept extended and a lateral view with the elbow flexed to 90° with the forearm in the mid-prone position. However, this is not always possible due to pain or deformity, hence leading to inadequate X-rays whose interpretation is dependent upon one’s experience.

**Figure 3:**
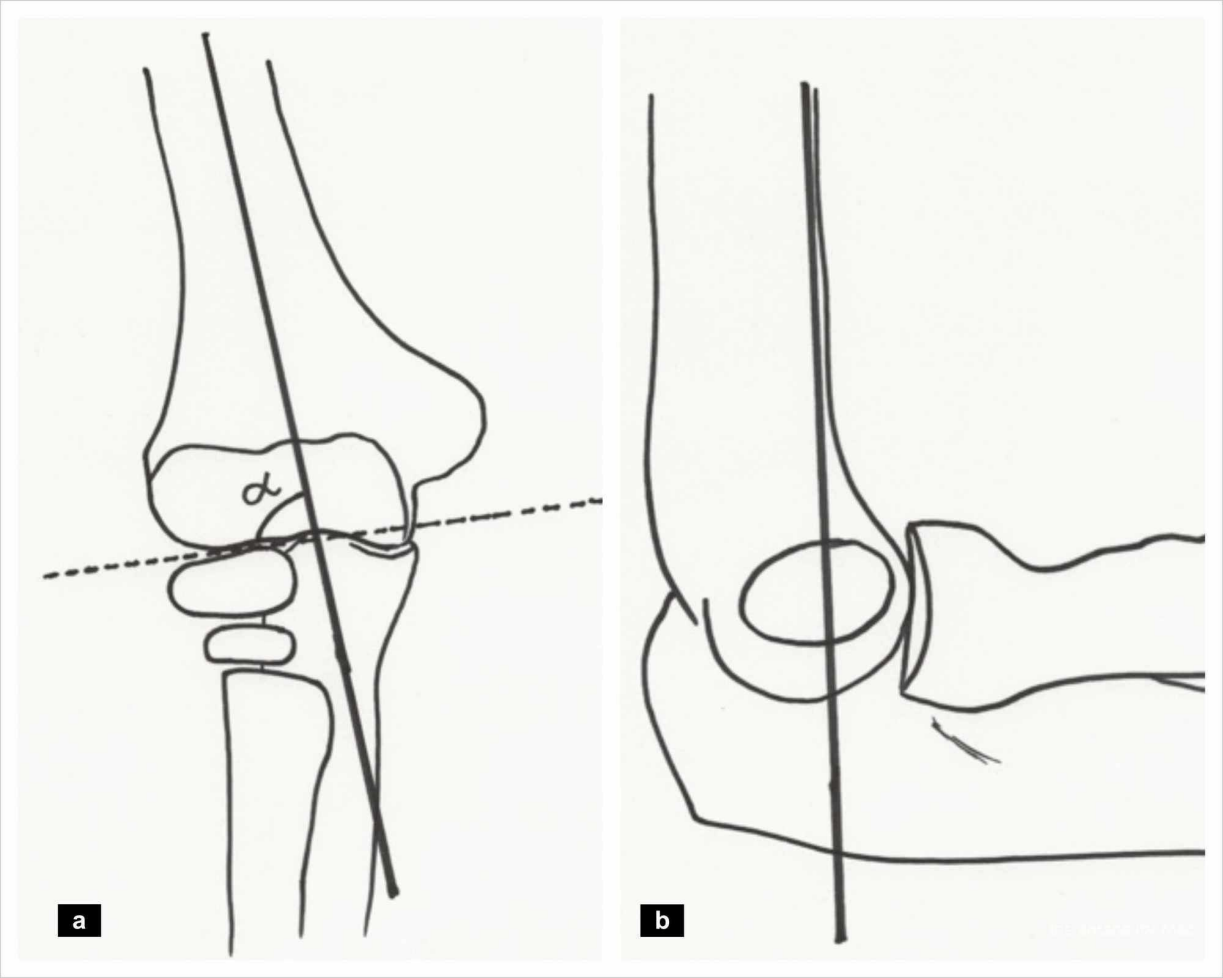
Radiographic evaluation (a) Baumann angle (alpha angle), and (b) Anterior humeral line. (Illustration courtesy P. M. Shenoy)

On the anteroposterior view, the angle between the capitellar physis and the long axis of the humerus, or the Baumann angle is noted (Figure 3a). The mean Baumann angle is 72° (range 64°-81°) [12]. An increase compared to the opposite side indicates a cubitus varus deformity. Translation of the distal fragment can also be appreciated in this view.

On the lateral view, particularly in undisplaced fractures, one may appreciate lucent shadows on the anterior and/or posterior aspect of the distal humerus known as the fat pad sign. The presence of a posterior fat pad sign is highly suggestive of an occult fracture in the elbow whereas an anterior fat pad sign alone can occur without a fracture [13,14].

The anterior humeral line is a line that is drawn along the anterior humeral cortex extended downwards to the capitellum in a true lateral view. It normally intersects the capitellum in its middle third [15]. In an extension type injury, this line frequently misses the capitellum and is a useful marker when determining the adequacy of fracture reduction during surgery. In Gartland type 2 injuries, there is some controversy as to when to consider surgical intervention particularly in the absence of rotational deformities. Iorio et al. have suggested to consider closed reduction and percutaneous pinning if the anterior humeral line passes anterior to the capitellum and is associated with a Baumann’s angle of more than 80° [16].

Management

A child presenting with a supracondylar fracture to the emergency department must be provided adequate pain relief and an above elbow back slab applied in a position of comfort. This is more important in completely displaced fractures where splinting may occasionally need to be done in near extension.

The Gartland classification assists in determining further management. Type 1 fractures and those elbows with a positive fat pad sign are treated conservatively in an above elbow cast with the elbow flexed to 90° if possible and neutral forearm rotation for 3-4 weeks. The treatment of type 2a fractures is generally controversial. Some centers choose to treat these fractures just by manipulation and above elbow cast. They need close observation to ensure further displacement does not occur [17]. Others choose to treat these by closed reduction and pinning particularly when the anterior humeral line is anterior to the capitellum and the Baumann angle is more than 80° [16]. Type 2b, type 3a and 3b are generally treated with closed reduction and pinning. BOAST guidelines recommend fixing these fractures using 2 mm K-wires whenever possible [3]. BOAST guidelines also recommend removal of these K-wires at three to four weeks [3].

Traditionally, type 3 fractures were treated as an urgent procedure soon after admission irrespective of whether it was day or night. BOAST guidelines suggest that night-time operating is not necessary unless there is no pulse, or clinical signs are suggestive of impaired hand and finger perfusion and evidence of threatened skin viability is present [3]. Gupta et al. have shown that there was no difference in either perioperative complications or an increased need for open reduction even in patients who had surgery more than 12 hours after injury [18].

Elevated straight arm skin traction has been used to treat these fractures in children less than 10 years of age quite effectively. Gadgil et al. treated 112 children with this technique and demonstrated excellent outcomes in 63% and poor outcomes were reported in only 2.6% of children [19]. This treatment has also been shown to be a viable option in low and middle income countries where surgical expertise and resources are limited [20].

Fracture Reduction Technique

This should be done under general anaesthetic and with C arm control for best results. Longitudinal traction is applied first with the forearm supinated to dislodge the fracture and this helps gain length and correct rotation. This may not be possible if there is a brachialis sign or if the proximal fragment spike is felt subcutaneously indicating buttonholing through the brachialis muscle causing interposition. In these cases, a “milking manoeuvre” has been described where under gentle traction, the anterior musculature is grasped proximally between the thumb and the fingers followed by gentle lateral pressure distally. This should be attempted not more than two times and a successful manoeuvre is indicated by a sudden release or occasionally an audible ‘pop’ [21]. Following this, medial or lateral translation and angulation is corrected. A flexion reduction manoeuvre is then performed with pressure of the surgeon’s thumb applied over the tip of the olecranon as the elbow is flexed. The elbow is then kept hyperflexed with the forearm pronated to lock the reduction [22].

A Jones anteroposterior view is obtained in the flexed elbow position along with slight oblique views to visualize reduction of the medial and lateral columns. A lateral view is obtained by externally rotating the arm in stable fractures. If the fracture is unstable, then the C arm should be rotated to obtain a lateral view to avoid the risk of losing a tenuous reduction.

Open Reduction Technique

Open reduction is indicated to obtain satisfactory alignment if closed reduction is unsuccessful. This is commonly due to soft tissue or neurovascular interposition. Mangat et al. showed in their case series that all patients with median or AIN injury associated with the supracondylar fracture were found to have either the nerve or vessel or both entrapped at the fracture site and suggested that patients presenting with these associated nerve injuries should be explored early [9]. This has however been disputed by Harris et al. who found that 97% of patients in their cohort had complete resolution of nerve palsy in spite of 70% having undergone a closed reduction and pinning [23]. It is estimated that up to 8% of supracondylar fractures need an open reduction [24].

Several approaches have been used in supracondylar fracture surgery including anterior, medial, lateral and posterior approaches. The decision to operate from a medial or lateral approach should be based on where the periosteal hinge is torn. Hence it is recommended to adopt a lateral approach in posteromedial displacement and a medial approach in posterolateral displacement [25].

The anterior approach with a transverse incision in the antecubital fossa is recommended as it can be extended proximally and distally easily. Access to the neurovascular structures is easy and is the approach of choice when considering vascular exploration and repair. The brachialis muscle is commonly torn and the fracture reduces easily under direct vision once the muscle is retracted. It has been shown that the anterior approach is safe, simple and easy to perform with good results and less incidence of loss of fracture reduction when compared to either a lateral or combined medial and lateral approach [26].

Thickness of K-Wires

BOAST guidelines recommend the usage of 2-mm K-wires where possible, to achieve stability [3]. Although 1.6-mm K-wires are commonly used to stabilize these fractures, it has been shown that larger diameter pins are far more stable compared to using 1.6-mm pins in the pin configurations tested in laboratory conditions. These included crossed pins and lateral divergent pins [27].

It has also been suggested that 2-mm K-wires are helpful in achieving a bicortical fixation in high oblique fractures even when angled at 74° to the horizontal. The 1.6-mm K-wires could only manage a maximum angulation of 68° beyond which the wire failed to engage the distal cortex and ended up being intramedullary with purchase only at the near cortex [28].

K-Wire Configuration

Several different configurations of pin placement have been described in the literature. They vary from crossed pin to lateral pin configuration to several combinations of both with no differences in terms of loss of fracture reduction [29].

**Figure 4:**
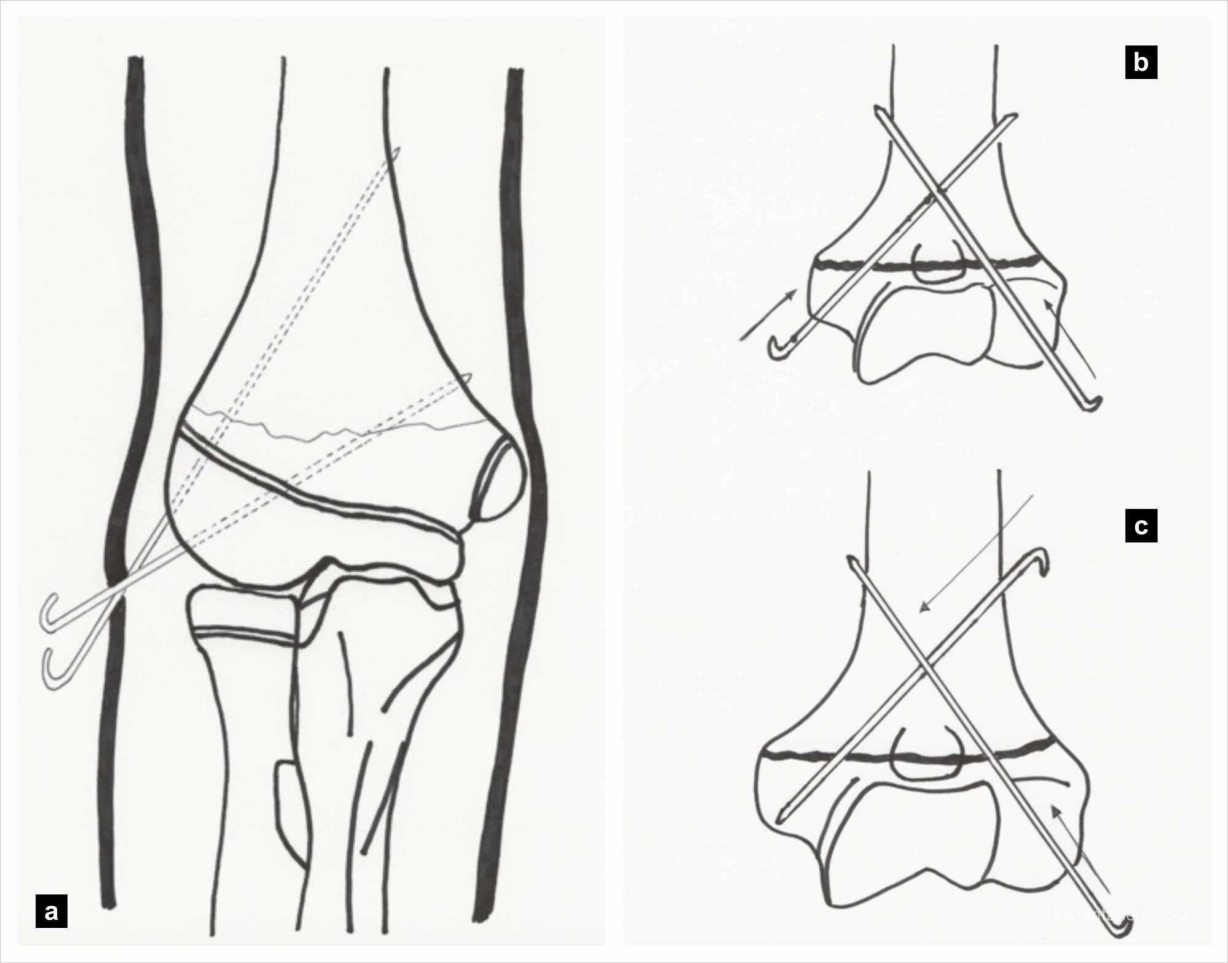
K-wire configuration for fixation (a) Lateral divergent technique, (b) Cross K-wiring technique, and (c) Dorgan’s technique. (Illustration courtesy P. M. Shenoy)

The use of a medial sided pin is associated with an increased risk of injury to the ulnar nerve in 8% of patients [30]. This may be due to a tethering effect within the cubital tunnel rather than direct nerve injury [31]. If a medial pin is used, techniques used to avoid ulnar nerve injury should be used and meticulously documented (Figure 4a) [3]. Fractures managed with lateral only K-wires did not pose any iatrogenic risk to the ulnar nerve either by conventional divergent lateral wiring or by Dorgan’s technique (Figure 4b) [32,33]. Dorgan’s technique is a cross wire fixation where the lateral wire is placed through the lateral condyle as in other procedures, but the second wire is placed from the lateral side starting in the proximal fragment and aimed medially and distally (Figure 4c) [32]. Biomechanical studies have shown superior fracture stability when a crossed pin construct is used compared to divergent lateral pin construct [27].

More than two wires are sometimes used particularly when fractures are unstable either with three lateral wires or three to four wires in cross wire configuration. It has been shown that fracture reduction is maintained better with three wires in crossed configuration (two lateral and one medial wire) and had a smaller change in Baumann angle compared to those fractures treated with other pin configurations [34].

Approach to a Pink and Perfused Hand

These injuries need urgent assessment and fracture reduction in theatre. Associated median or anterior interosseous nerve palsy could indicate brachial artery injury as these structures are in close proximity and may require open reduction and exploration of the neurovascular structures. There is lack of consensus of how to manage these patients.

**Figure 5:**
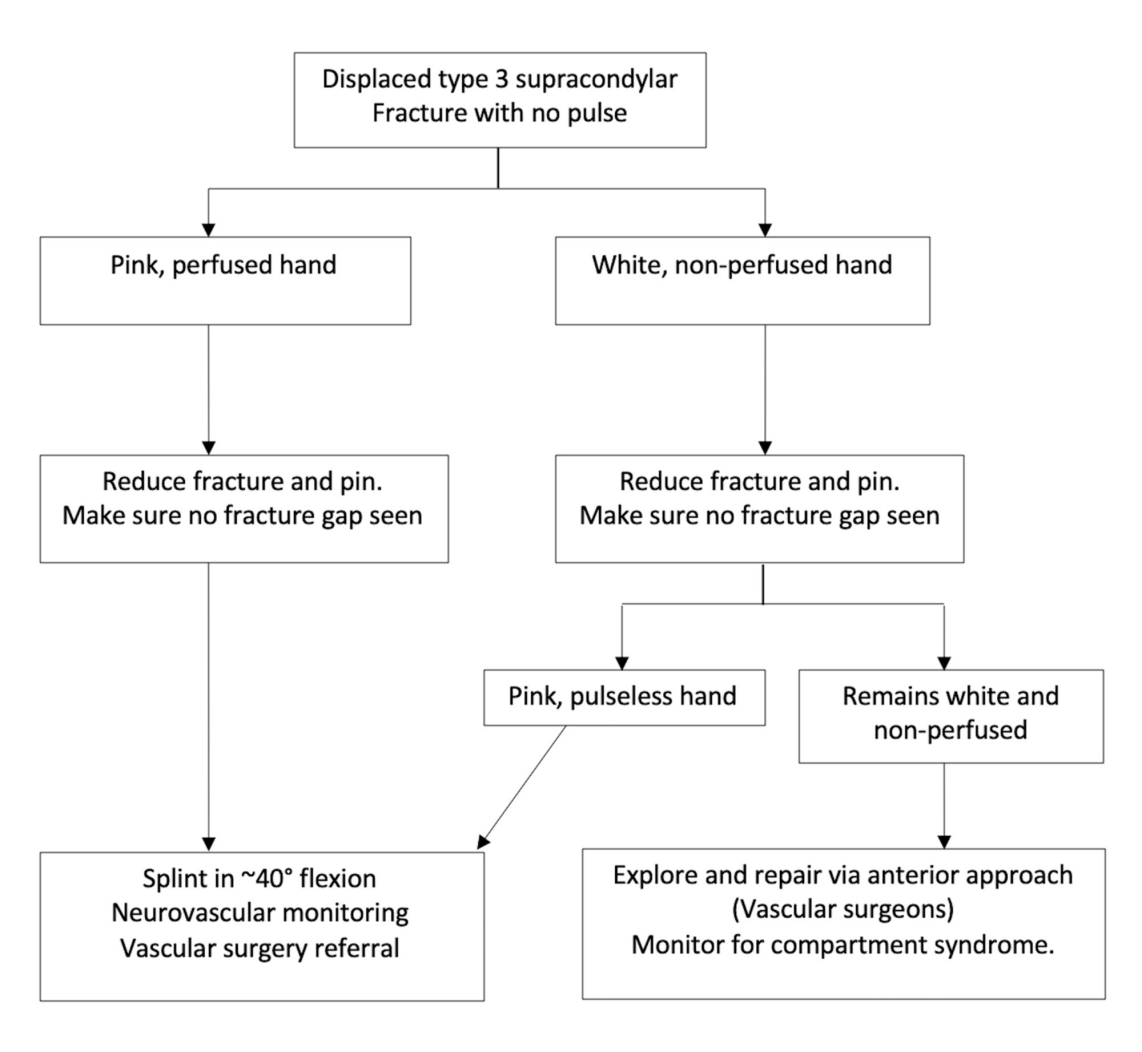
Treatment algorithm for managing a displaced supracondylar fracture in a child with no pulse Algorithm by P. M. Shenoy

The aim of treatment is to obtain a closed reduction without any gap at the fracture site followed by pinning of the fracture. A gap at the fracture site could be due to soft tissue interposition which can include neurovascular structures. In most cases, the pulse returns immediately after a successful reduction. The fracture can then be pinned percutaneously. Open reduction may be needed in complex fracture patterns or if the fracture is unstable or irreducible with a gap at the fracture site. As long as the hand remains well perfused at the end, a splint is applied in about 40° flexion and the child is monitored closely to ensure there is no deterioration in neurovascular status. An early vascular surgical consultation can be useful to avoid any delays in deciding to proceed with an exploration if there are any concerns (Figure 5).

Approach to a White and Non-Perfused Hand

Children presenting with a white and non-perfused hand should be treated as an emergency. Focus should be to try and reduce the fracture as soon as possible to see if the perfusion of the hand improves. If the hand becomes pink and perfused, then the approach suggested previously can be followed. If it remains white, open exploration and if needed repair via an anterior approach with the vascular surgeons should be undertaken (Figure 5). Compartment syndrome can develop in children who undergo a successful vascular repair and hence fasciotomies may need to be considered [35].

Other complications

Complications commonly encountered with these fractures include pin migration, pin site infection, malunion including malrotation (gunstock deformity) leading to abnormal carrying angle, neurovascular injury, compartment syndrome and reduced range of motion. Sinikumpu et al. also report having found isolated cases of undisplaced type 1 fractures which resulted in a change in carrying angle in the long term which suggests that there is growth disruption due to the energy of trauma irrespective of the original displacement [36].

Cubitus Varus

This is the most common complication following a supracondylar fracture. It does not usually cause any problems with elbow range of motion and is painless. A study by Labelle et al. showed no functional differences in children treated with or without surgical correction of cubitus varus suggesting the surgery was purely to improve cosmesis [37].

O’Driscoll et al. have, however, suggested that a cubitus varus deformity may lead to a tardy posterolateral instability two to three decades post injury in adulthood. They speculate this is due to medial deviation of the mechanical axis, triceps line of pull and the olecranon leading to increased external rotation torque on the ulna. This then leads to a stretching and attenuation of the lateral ligament complex causing instability. They have hence suggested that cubitus varus deformity is not a benign condition and recommended treatment of this problem with corrective osteotomy and ligament reconstruction in symptomatic adults [38].

Stiffness

Stiffness is seen in most patients with a supracondylar fracture which is quite noticeable after removal of the plaster cast. We know from experience that full range of motion is usually restored with time. Ducic et al. have shown that children who had physiotherapy showed faster improvement in the range of motion in the first few months compared to those children who didn’t. However, after 12 months there was no difference in range of motion in the two groups suggesting that physiotherapy is not routinely recommended in patients with supracondylar fractures [39].

## Conclusions

Supracondylar fractures in children are common but can be distressing injuries to the child, parents and the surgeon. Neurovascular assessment is of paramount importance and must be documented in detail. It is critical to determine hand perfusion to determine the urgency of treatment. Controversy continues to exist with continuously evolving literature on how these are best managed with regards to pin size and configuration. The BOAST guidelines aim to standardise treatment across the UK and globally and is useful to incorporate into one’s management protocol to provide good practice based on the current evidence.

## Figures and Tables

**Figure 6:**
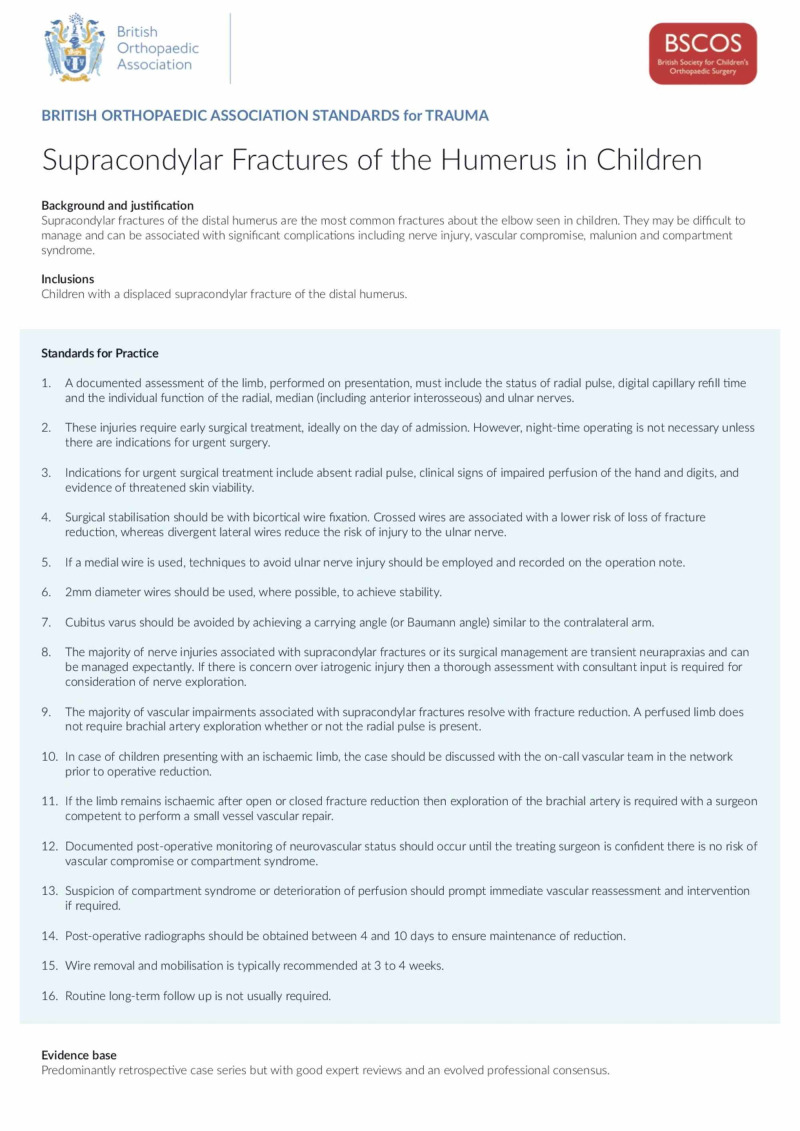
BOAST guidelines- Supracondylar fractures of the humerus in children Included with permission [3]. Any follow-up questions with regards to the BOAST guidelines can be emailed to
